# The effect of trial repetition and problem size on the consistency of decision making

**DOI:** 10.1371/journal.pone.0216235

**Published:** 2019-05-06

**Authors:** Vladimír Bureš, Daniela Ponce, Pavel Čech, Karel Mls

**Affiliations:** Dept. of Information Technologies, University of Hradec Králové, Rokitanského 62, Hradec Králové, 50003, Czech Republic; Shandong University of Science and Technology, CHINA

## Abstract

Human decision making involving many alternatives is encumbered with inconsistent prioritization. Although inconsistency is assumed to grow with the number of comparisons, it is shown to be reduced by conscious awareness under certain conditions. This study experimentally investigated the effect of repeating a criteria ranking task on inconsistency scores as measured by four different inconsistency coefficients. A total of 107 participants were engaged in a selection task that comprised of ranking from 3 to 10 criteria and was repeated in three trials. Upon completing the first trial, the participants were informed about the inconsistency issues and could improve their ranking in another two trials. The inconsistency score was computed for each set of comparisons and the effect of repeating the selection task on inconsistency concerning the number of criteria was analyzed using the repeated measures ANOVA. The results reveal a significant change in the inconsistency as the task was repeated but the difference depended on the number of criteria. There exists a borderline in the problem size under which the rankings are associated with significantly lower inconsistency, while the rankings with the larger number of criteria were found to have significantly higher inconsistency.

## Introduction

In the task of multicriteria alternative selection, the individual has to rank alternatives from a finite set according to multiple criteria. This relatively simple task represents truly multidisciplinary phenomenon. From either methodological or application perspective, it represents one of the most important and popular topics in various disciplines. Methodological and fundamental issues are investigated in operational research [1], decision sciences [2], psychology [3] or computer science [4]. Application domains are countless, ranging from tourism [5] or environmental issues [6, 7] to engineering [8] and energetic systems [9, 10]. The pairwise comparison method is applied because it is much easier for people to assess two alternatives at a time than handling all of them at once. This assumes that all of the alternatives are compared in pairs. Then, by using an appropriate algorithm, the overall ranking is synthesized. Several models and methods have been developed to aid this task. A common method is to assign preferences to alternatives [2]. Once the pairwise comparisons of priorities are determined, a priority vector of alternatives can be derived and used for final alternatives ranking [11].

However, pairwise comparison is associated with inconsistencies. When comparing *n* priorities, a set of (*n* − 1) basic comparisons can be defined such that the values of all pairwise comparisons ((n*(*n* − 1)/2) in total) can be consistently derived from the values of these basic comparisons. Because the method of priority comparison specification requires the decision maker to assign values to all pairwise comparisons of priorities, it is mostly impossible to produce perfectly consistent complete priority comparisons in practice. Moreover, it is expected that inconsistency will rise with an increasing number of comparisons [12–14]. The inconsistency in priorities comparison also rises due to mistakes made by decision makers [15] and because they are not certain in their judgements [11], they do not understand the decision context or they do not check priority comparisons for consistency [16]. In decision theory, typical inconsistencies are intransitivities or violations of monotonicity or reversals of preferences. Some models of decision making e.g. regret theory [17] predict intransitivities for instance. If they occur, they may be due to mistakes or to the use of some specific heuristic/decision rules. Other models predict violations of stochastic dominance or preference reversals. Their models view decisions as intrinsically stochastic. Reversals of preferences then reflect uncertainty of the decision maker or incompleteness of preferences or a preference for randomization. Finally, most empirical work on decisions still include a form of noise (or so called trembling hand) on top the decision rules used.

There are several methods of inconsistency quantification—see e.g. [18, 19] and they allow us not only to decide on the acceptability of inconsistent alternative comparison matrix but also to compare the measure of the inconsistency of several matrices (given by different experts). Inconsistency measures may be based on ordinal and also cardinal comparisons of alternatives. Some of them make use of parameters calculated from a large set of randomly generated comparison matrices [14, 20, 21]. These methods differ in behavior, degree of the resemblance to other inconsistency indices and in ease of calculation [22]. Studies comparing these methods evaluate inconsistency quantification methods for different sets of comparison matrices with numerical values according to some chosen criteria [13, 23, 24].

The existing research gap is associated with two groups of experts coping with inconsistency. First, properties of the inconsistency quantification methods and comparison matrices are explored from the computer science perspective [25–28]. These papers are either purely theoretical or empirical studies. The issue is that apart from scarce exceptions, all of the studies that have been referred to so far make use of randomly generated comparison matrices. To our knowledge, the only two studies devoted to the inconsistency of empirically obtained alternative comparison matrices are a demonstrative experiment [29] and a regular experimental study [30].

Second, there are some research works based on empirical studies dealing with investigation of the phenomenon of inconsistency in human manifestation from the psychological point of view [31–35]. The issue is that these works are not focused on the multicriteria decision making of individuals and they do not make use of a numerical measure that allows ordinal or even cardinal comparison. Therefore, we explore inconsistency from the interdisciplinary perspective because it is both psychological phenomenon that is associated with human cognitive abilities and also a computer science problem that has to be addressed and tackled in relation to formal representation and quantitative analysis of multiattribute decision-making task.

Concerning these issues, we have addressed two questions in this study. First, we ask how and to what extent inconsistency changes in repeated solving of the same task of multicriteria decision making? Second, we ask does the inconsistency of the multicriteria choice making task change when the size of the task is modified?

## Materials and methods

### Participants and materials

The data gathering process was initiated by a call for participation that was issued by the authors at the university settings. Altogether, 198 students studying either information management or applied informatics study programme enrolled in the experiment and participated in the data gathering process. At the outset of the experiment, the students were informed about both the voluntary nature of their participation in the study, and the possibility to opt out at any time. During the study no personal data was processed and data collection represented a part of the course curriculum, therefore, the Committee for Research Ethics at the University of Hradec Králové did not require any special consent to participate in the study. Since all of the subjects represented a heterogeneous group of individuals, the topic suitable for the evaluation had to be carefully considered due to the necessity to find a domain which would have been understandable and familiar to all subjects. Eventually, the subjects were presented with a simulated decision-making task of car selection with a list of at most ten criteria for a comparison of the alternatives. These criteria were: acquisition price, bodywork colour, car maker, average consumption, engine capacity, maximal speed, interior equipment, acceleration, service availability, and parking assistant. This allowed us to ensure a certain level of homogeneity of subjects from the perspective of the decision-making task. Thus, for this study, all of the subjects can be considered as equally competent for evaluation.

### Applied measures

We used four inconsistency measuring methods, named: Consistency Index (CIndex), Consistency Ratio (CRatio), Euclidean Distance (EDA) and Euclidean Normalized Distance (EDANorm). In the following definitions, we assume that *A* = [*a*_*ij*_] is a multiplicative priority comparison matrix of dimension *n*.

The CIndex was defined by [18] as

$$\begin{array}{c} CIndex\left(A\right)=\frac{\lambda_{max}-n}{n-1} \end{array}$$ (1)

where λ_*max*_ is the principle eigenvalue of *A*; *CIndex* ≥ 0.

The CRatio is a standardized version of the CIndex. CIndex is divided by a real number RI where RI is calculated as average CIndex of a very large number of randomly generated reciprocal matrices of size *n*:

$$\begin{array}{c} CRatio\left(A\right)=\frac{CIndex(A)}{RI} \end{array}$$ (2)

EDA is then defined as

$$\begin{array}{c} EDA\left(A\right)=\sqrt{\sum_{i,j\in N}{\left(b_{ij}-v_{ij}\right)}^{2}} \end{array}$$ (3)

where *B* = [ln *a*_*ij*_], $w_{i}=\frac{1}{n}\sum_{i\in N}b_{ij}$ is an arithmetic mean weight vector and *V* = [*v*_*ij*_] = [*w*_*i*_ − *w*_*j*_].

Finally, the EDANorm is a normalized version of EDA and it is calculated as

$$\begin{array}{c} EDANorm\left(A\right)=\frac{EDA(A)}{\sqrt{\sum_{i,j\in N}b_{ij}^{2}}} \end{array}$$ (4)

### Procedure

**Fig 1:**
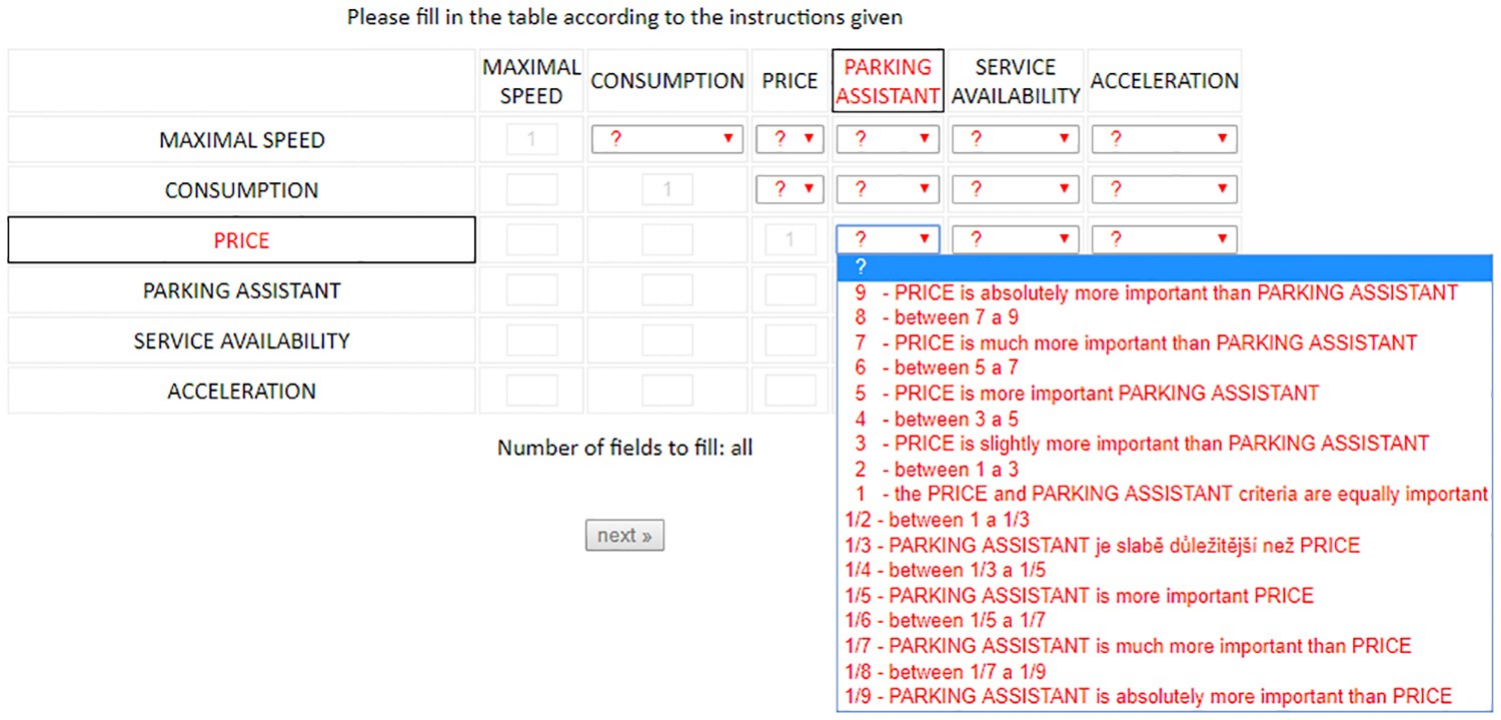
Screen image of the data gathering application. The screen image shows the user interface of the data gathering application with compared alternatives and a list of values specifying the user preferences.

The experiment took place in a dedicated computer lab located on the university campus. A proprietary web-based application was built based on PHP, HTML, CSS, JavaScript, and MySQL technologies. This web application gathered, checked and saved data, measured time spent with evaluation of alternatives criteria comparison, and dealt with proper formatting to provide a user-friendly interface (see Fig 1). Reckoning of the eigenvalues was performed in a console application developed in the C# programming language. The core of this application was focused on input acquirement and output formatting. Reckoning itself was based on the third-party public domain licensed library provided by Codeproject (Simple Matrix Library for.net, URL https://www.codeproject.com/Articles/5835/DotNetMatrix-Simple-Matrix-Library-for-NET).

The acquired results were double-checked with the existing literature [36].

There were no time limitations associated with the evaluation process. The subjects were only allowed to participate in the study once. There were three repeated rounds (hereafter referred to as trials) with eight steps, which represented mutually evaluated matrices with dimensions from three to ten (hereafter referred to as problem size). Because it is dominant in the field from the long-term perspective, the evaluation was based on the analytic hierarchy process (hereafter referred to as AHP) that was developed by [18], in which ranging from 1 to 9 and their corresponding inverse values are used. Alternatives′ criteria were randomly chosen and situated in the matrix in every trial and every problem size in other to avoid the memory effect. The subjects were given a comparison matrix of all cells at once (as opposed to cell-by-cell) and they were allowed to re-edit once they had entered comparison values prior to the submission of entire comparison matrix. At the beginning of the first trial, the subjects were only informed about the activity without mentioning the main aim and purpose. Only introductory information was provided, such as Saaty’s method, or evaluated car specifications. At the beginning of the second trial, the concept of inconsistency was explained and subjects were asked to minimize inconsistency during their evaluation of alternatives. The last trial was performed without any additional information being provided.

### Statistical analysis

The acquired data were cleaned to obtain a coherent dataset with consistent and complete data associated with each subject. Therefore, unfinished, incomplete or incorrectly conducted evaluations from 91 subjects were excluded from the dataset. The remaining 107 subjects created a dataset with 2,568 comparison tasks. The statistical analyses were conducted with R and IBM SPSS statistical software packages. Repeated measures ANOVA was carried out to compare outcomes of different inconsistency coefficients in respect to the problem size and trial. Repeated measures ANOVA allowed a set of inconsistency scores to be related across trials and problem sizes provided by the same participant. Thus, the inconsistency coefficients calculated for each problem size level and trial level were considered as within subject factors. The repeated measures ANOVA assumes that there are approximately equal variances between each pair of scores in levels of repeated variables. This is referred to as sphericity. The sphericity assumption was tested with Mauchly’s test. The multivariate test results were reported in case the assumption of sphericity was violated. As reported by [37] the multivariate procedure is more powerful if the violation of sphericity and the sample size are both large. The post hoc test with pairwise assessments of experimental conditions are based on the Bonferoni adjustment. The Bonferoni test is regarded as robust in terms of Type I error under the conditions of non-sphericity [38]. Mean differences (M) and corresponding 95% confidence intervals (CI) were also reported. Due to different scales of each coefficient, the data were normalized to N(0,1). To uncover a pattern in the inconsistency scores related to number of comparisons made, problem sizes were aggregated into two groups. The aggregation was executed with mean as the aggregation function. Repeated measures ANOVA was reapplied to confirm patterns revealed in the first stage.

## Results

The results of statistical tests with p-value < 0.05 were reported as statistically significant. The effect size was measured by the partial eta squared statistics. The partial eta squared statistics can be interpreted as the amount of variance explained by the independent variable. According to [39] the indicative effect sizes are 0.01 = small effect, 0.06 = medium effect, 0.14 = large effect.

### Effect of number of trials on inconsistency with respect to problem size

**Fig 2:**
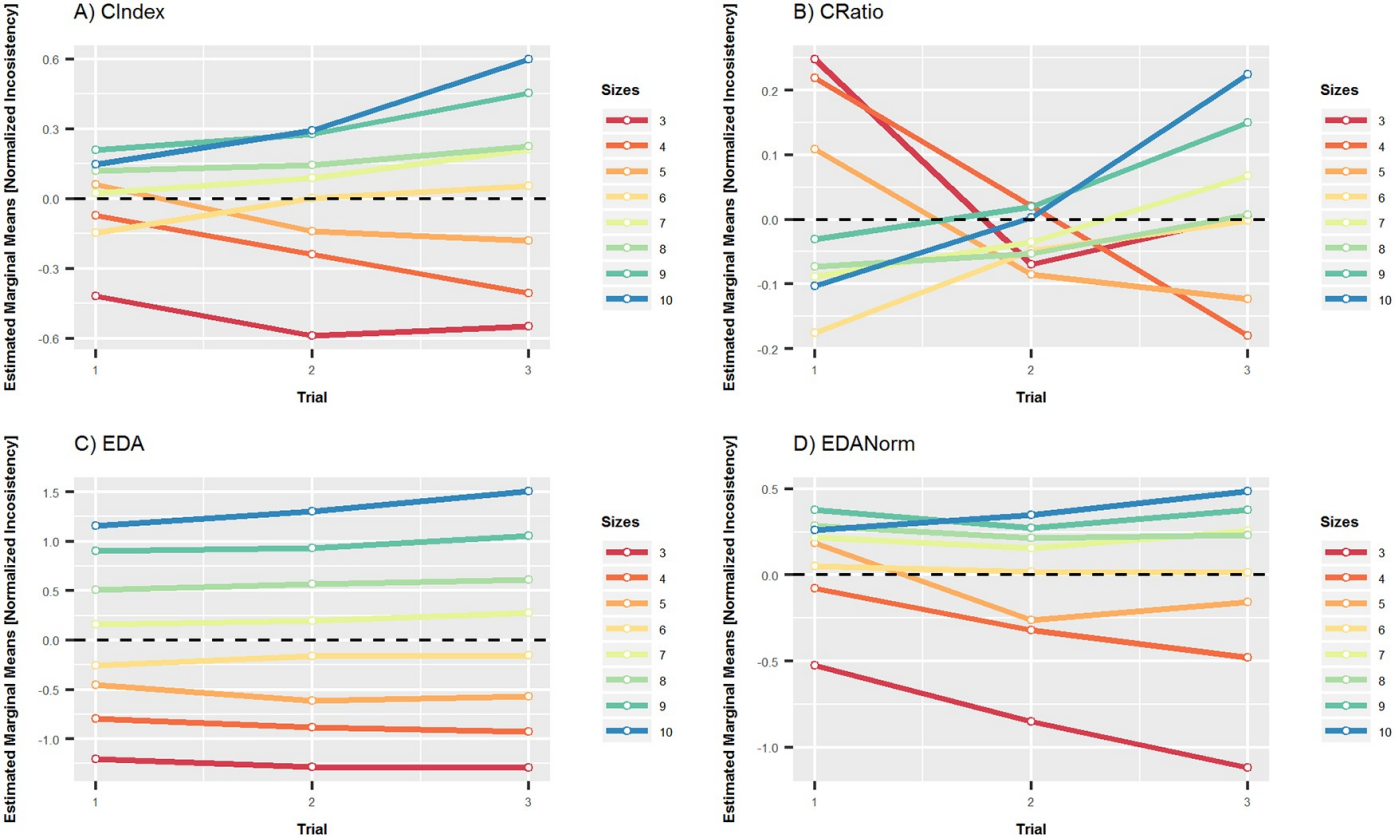
Estimated marginal means of normalized inconsistencies with respect to the problem size. Estimated marginal means of normalized inconsistencies across trials and problem sizes measured by four coefficients. The 95% confidence intervals are omitted due to clarity and y-axis scales are different in each subplot not to flatten the trend in data.

Repeated measures ANOVA has been conducted to assess the effect of repeating the decision making problem on the level of inconsistency achieved in regard to the problem size. The trial number (1–3) is considered as a within subject factor, which is further decomposed in respect to the problem size. The results show that there is a significant interaction between the trial and problem size Wilks’ Lambda = 0.678, F(14, 93) = 3.161, p < 0.001, partial eta squared = 0.322 (large effect). This indicates that the effect of repeating the decision making task on inconsistency changes across different problem sizes. The interaction is plotted in Fig 2 for each coefficient separately. Fig 2 reveals that the inconsistency increases with trial for larger problem sizes.

The pairwise comparison with Bonferoni adjustment confirms a statistically significant increase in inconsistency between the first and third trial, and also between the second and third trial for problem size 10, as measured by all coefficients (see Table 1).

**Table 1:** Differences in estimated marginal means of normalized inconsistency between trials for problem size 10. The positive mean difference indicates an increase in the inconsistency between trials. The negative indicates a decrease in the inconsistency between trials.

Coefficient	trial	trial	Mean Difference	Std. Error	Sig.	Lower Bound	Upper Bound
CIndex	2	1	0.062	0.068	0.367	-0.197	0.073
	3	1	0.308	0.088	0.001	-0.482	-0.134
		2	0.246	0.074	0.001	-0.393	-0.100
CRatio	2	1	0.045	0.049	0.367	-0.142	0.053
	3	1	0.223	0.063	0.001	-0.349	-0.097
		2	0.178	0.053	0.001	-0.284	-0.072
EDA	2	1	0.064	0.053	0.226	-0.169	0.040
	3	1	0.232	0.061	0.000	-0.352	-0.112
		2	0.168	0.050	0.001	-0.267	-0.068
EDANorm	2	1	0.071	0.068	0.296	-0.206	0.063
	3	1	0.207	0.071	0.004	-0.347	-0.066
		2	0.135	0.057	0.019	-0.248	-0.023

There was also a decrease in inconsistency between first and second trial for problem size 3. This decrease is statistically significant for CIndex, CRatio and EDANorm but no for EDA (see Table 2).

**Table 2:** Differences in estimated marginal means of normalized inconsistency between trials for problem size 3. The positive mean difference indicates an increase in the inconsistency between trials. The negative mean difference indicates a decrease in the inconsistency between trials.

Coefficient	trial	trial	Mean Difference	Std. Error	Sig.	Lower Bound	Upper Bound
CIndex	2	1	-0.207	0.097	0.036	0.014	0.400
	3	1	-0.116	0.141	0.415	-0.164	0.395
		2	0.091	0.110	0.406	-0.309	0.126
CRatio	2	1	-0.385	0.181	0.036	0.026	0.743
	3	1	-0.215	0.262	0.415	-0.305	0.735
		2	0.170	0.204	0.406	-0.574	0.234
EDA	2	1	-0.065	0.037	0.085	-0.009	0.140
	3	1	-0.057	0.042	0.180	-0.027	0.141
		2	0.008	0.035	0.815	-0.078	0.062
EDANorm	2	1	-0.369	0.195	0.062	-0.018	0.755
	3	1	-0.500	0.200	0.014	0.103	0.897
		2	-0.131	0.166	0.431	-0.198	0.461

There was a decrease in inconsistency between first and second trial for problem size 5 (see Table 3). However, this decrease is statistically significant just for EDA and EDANorm. A decrease in inconsistency between third and first trial is statistically significant for EDANorm only.

**Table 3:** Differences in estimated marginal means of normalized inconsistency between trials for problem size 5. The positive mean difference indicates an increase in the inconsistency between trials. The negative mean difference indicates a decrease in the inconsistency between trials.

Coefficient	trial	trial	Mean Difference	Std. Error	Sig.	Lower Bound	Upper Bound
CIndex	2	1	-0.151	0.155	0.332	-0.458	0.156
	3	1	-0.139	0.118	0.244	-0.373	0.096
	3	2	0.012	0.144	0.931	-0.272	0.297
CRatio	2	1	-0.145	0.149	0.332	-0.441	0.15
	3	1	-0.133	0.114	0.244	-0.359	0.093
	3	2	0.012	0.138	0.931	-0.262	0.286
EDA	2	1	-.118	0.049	0.019	-0.215	-0.02
	3	1	-0.08	0.046	0.085	-0.171	0.011
	3	2	0.038	0.047	0.428	-0.056	0.132
EDANorm	2	1	-0.233	0.118	0.05	-0.467	0
	3	1	-.219	0.101	0.033	-0.42	-0.018
	3	2	0.014	0.121	0.908	-0.227	0.255

In the case of problem size 6, there was no statistically significant increase/decrease in inconsistency between trials for any indices used (see Table 4).

**Table 4:** Differences in estimated marginal means of normalized inconsistency between trials for problem size 6. The positive mean difference indicates an increase in the inconsistency between trials. The negative mean difference indicates a decrease in the inconsistency between trials.

Coefficient	trial	trial	Mean Difference	Std. Error	Sig.	Lower Bound	Upper Bound
CIndex	2	1	0.035	0.093	0.705	-0.148	0.219
	3	1	0.157	0.111	0.161	-0.063	0.376
	3	2	0.121	0.093	0.193	-0.062	0.305
CRatio	2	1	0.031	0.08	0.705	-0.129	0.19
	3	1	0.136	0.096	0.161	-0.055	0.327
	3	2	0.106	0.081	0.193	-0.054	0.265
EDA	2	1	0.001	0.047	0.987	-0.092	0.093
	3	1	0.049	0.052	0.349	-0.054	0.151
	3	2	0.048	0.042	0.259	-0.036	0.131
EDANorm	2	1	-0.087	0.092	0.347	-0.27	0.096
	3	1	-0.041	0.094	0.663	-0.227	0.145
	3	2	0.046	0.083	0.582	-0.119	0.211

Interaction between the trial and problem size shows different inconsistency patterns for different pairs of problem sizes. In the case of problem size 3 and 10, the inconsistency increased from size 3 to size 10 in all but one cases (all trials, all indices). However, the inconsistency increase is statistically significant in all three trials for CIndex, EDA and EDANorm only. In the case of CRatio index, the inconsistency increase is not statistically significant, and more over, for the trial one, a statistically significant inconsistency decrease is shown (see Table 5). In the case of problem size 5 and 6, the inconsistency increased from size 5 to size 6 in 9 cases and it decreased in 3 cases (cases of all three trials and all four indices). However, a statistically significant increase in inconsistency from size 5 to size 6 was only found for EDA index for all three trials and in the trial 3 also for CRatio and EDANorm indices (see Table 6).

**Table 5:** Differences in estimated marginal means of normalized inconsistency between sizes 10 and 3. The positive mean difference indicates an increase in the inconsistency with increased size. The negative mean difference indicates a decrease in the inconsistency with increased size.

Coefficient	trial	Mean Difference	Std. Error	Sig.	Lower Bound	Upper Bound
CIndex	1	0.564	0.113	0.001	0.34	0.788
	2	0.833	0.076	0.001	0.681	0.984
	3	0.987	0.119	0.001	0.752	1.223
CRatio	1	-.378	0.17	0.028	-0.714	-0.041
	2	0.052	0.077	0.503	-0.101	0.205
	3	0.06	0.182	0.742	-0.3	0.42
EDA	1	2.358	0.073	0.001	2.214	2.502
	2	2.488	0.071	0.001	2.347	2.629
	3	2.645	0.084	0.001	2.478	2.812
EDANorm	1	0.745	0.165	0.001	0.418	1.072
	2	1.182	0.123	0.001	0.938	1.426
	3	1.444	0.124	0.001	1.199	1.69

**Table 6:** Differences in estimated marginal means of normalized inconsistency between sizes 6 and 5. The positive mean difference indicates an increase in the inconsistency with increased size. The negative mean difference indicates a decrease in the inconsistency with increased size.

Coefficient	trial	Mean Difference	Std. Error	Sig.	Lower Bound	Upper Bound
CIndex	1	-0.094	0.101	0.352	-0.295	0.106
	2	0.092	0.133	0.493	-0.173	0.356
	3	0.201	0.131	0.129	-0.06	0.461
CRatio	1	-0.187	0.097	0.056	-0.378	0.005
	2	-0.011	0.125	0.931	-0.259	0.237
	3	0.083	0.12	0.49	-0.154	0.32
EDA	1	0.265	0.043	0.001	0.179	0.351
	2	0.383	0.045	0.001	0.294	0.472
	3	0.393	0.051	0.001	0.292	0.495
EDANorm	1	0.036	0.09	0.692	-0.142	0.213
	2	0.182	0.106	0.091	-0.029	0.393
	3	0.214	0.088	0.017	0.04	0.388

### Effect of number of trials on inconsistency with respect to aggregated problem sizes

The pattern covering decreasing inconsistency for small problem sizes and increasing inconsistency for larger problem sizes with repeating the decision making task was further analyzed by aggregating the problem sizes in two groups. The problem sizes were grouped based on the differences between the estimated marginal means of the first trial and the third trial in the CIndex for which the pattern was most pronounced. Table 7 shows the mean differences in inconsistency between trials across problem sizes; as it can be seen, after explanation of the inconsistency concept (between the trial one and two), a decrease in inconsistency occurred for small sizes (3 to 5) and size 8. One more trial did not lead to decrease in inconsistency as the inconsistency between the trials 2 and 3 decreased for size 4 only. Finally, when we compare trials 1 and 3, the decrease in inconsistency for sizes 3-5 can be still observed, but not for size 8. Apparently, even having in mind the knowledge of the inconsistency concept, the subjects were able to decrease the inconsistency for small sizes only, and the decrease was less notable with more trails completed (true for sizes 3 and 5, false for size 4). We can guess that the effect described is due to the anchoring effect combined with the fact that for each trial the order of decision-making criteria was generated randomly.

**Table 7:** Estimated marginal mean differences in normalized inconsistencies of the CIndex coefficient in between trials computed as (e.g. trial 2 score–trial 1 score). The negative mean difference refers to a decreasing inconsistency while the positive mean difference refers to an increasing inconsistency.

Size	Mean diff.		
	trial 2-1	trial 3-2	trial 3-1
3	-0.207	0.091	-0.116
4	-0.105	-0.159	-0.263
5	-0.151	0.012	-0.139
6	0.035	0.121	0.157
7	0.075	0.068	0.144
8	-0.032	0.116	0.084
9	0.096	0.114	0.210
10	0.062	0.246	0.308

**Fig 3:**
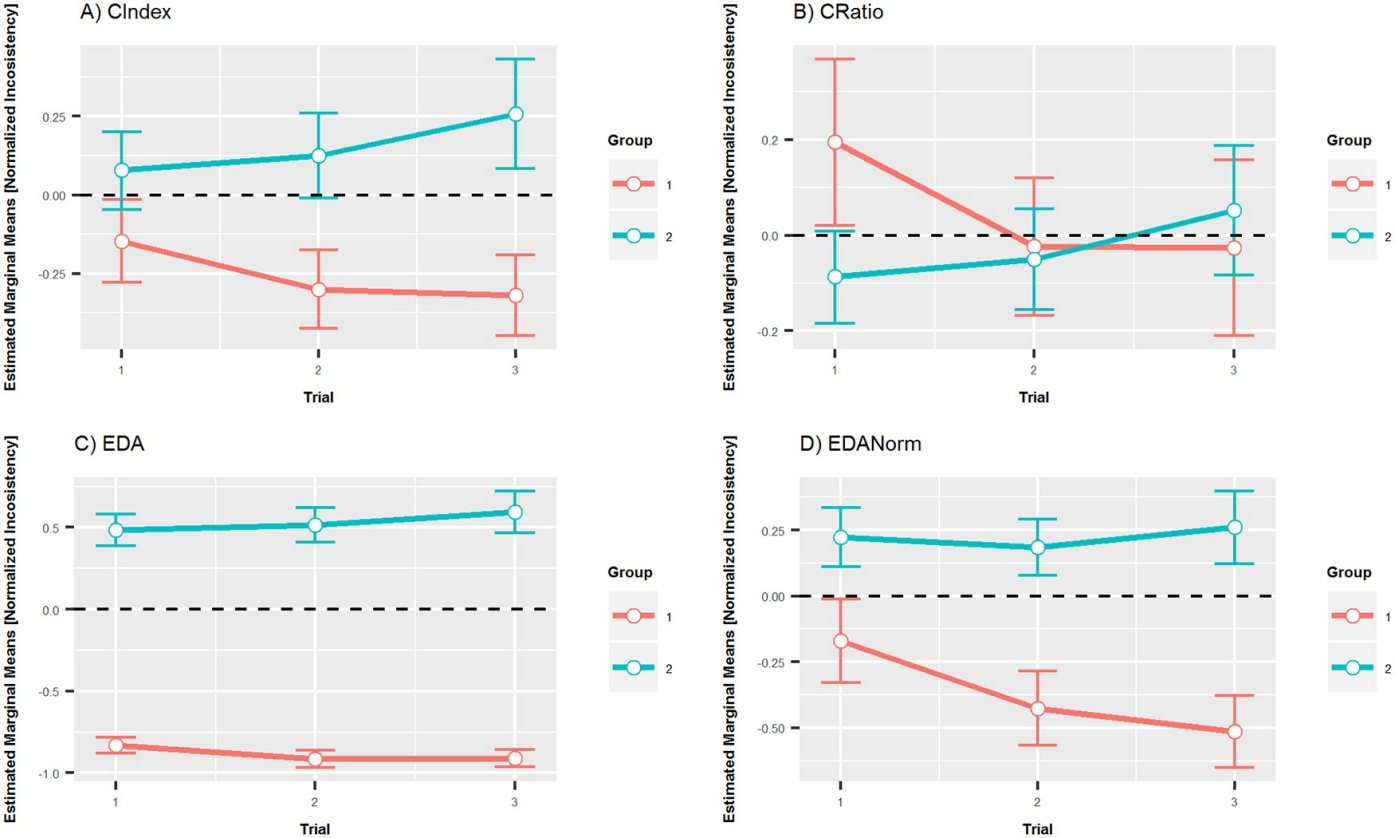
Estimated marginal means of normalized inconsistencies with respect to the grouped problem size. Estimated marginal means of normalized inconsistencies across trials and grouped problem sizes measured by four coefficients. There are different y-axis scales in each subplot not to flatten the trend in the data.

Thus, the groups were defined as follows: Group 1 (problem size 3–5), Group 2 (problem size 6–10). Repeated measures ANOVA has been performed to assess the effect of repeating the decision-making problem on the level of inconsistency achieved with problem sizes aggregated into two groups. The trial number (1–3) and the group (1–2) are considered as within subject factors. The results show that there is a significant interaction between the trial and group Wilks’ Lambda = 0.836, F(2, 105) = 10.284, p < 0.001, partial eta squared = 0.164 (large effect). This indicates that as the decision making task is repeated the inconsistency changes in different groups. The interactions are plotted in Fig 3.

The pairwise comparison with Bonferoni adjustment revealed that there is a significant decrease in inconsistency between the first and third trial in Group 1 and increase in inconsistency for Group 2 for CIndex coefficient. The same pattern can be observed for EDA coefficient. In the case of a CRatio coefficient, the decrease in inconsistency in Group 1 is significant between first and second trial but it is not significant between first and third. The increase in inconsistency measured by the CRatio coefficient in Group 2 is significant between first and third round. Concerning the EDANorm coefficient the decrease in inconsistency in Group 1 between first and third trial is significant. In Group 2, the increase in inconsistency between first and third trial is not significant (see Table 8).

**Table 8:** Differences in estimated marginal means of normalized inconsistency between trials and grouped sizes. The positive mean difference indicates an increase in the inconsistency with increased size. The negative mean difference indicates a decrease in the inconsistency with increased size.

Coefficient	Group	Trial	Trial	Mean Diff.	Std. Err.	Sig.	Lower Bound	Upper Bound
CIndex	1	2	1	-0.154	0.080	0.058	-0.314	0.005
		3	1	-.172	0.080	0.033	-0.331	-0.014
		3	2	-0.018	0.074	0.804	-0.164	0.128
	2	2	1	0.047	0.046	0.311	-0.045	0.139
		3	1	0.180	0.066	0.007	0.050	0.310
		3	2	0.133	0.047	0.006	0.040	0.227
CRatio	1	2	1	-.219	0.102	0.035	-0.421	-0.016
		3	1	-0.221	0.116	0.058	-0.451	0.008
		3	2	-0.003	0.098	0.978	-0.197	0.192
	2	2	1	0.037	0.037	0.316	-0.036	0.109
		3	1	0.139	0.051	0.008	0.037	0.241
		3	2	0.103	0.037	0.007	0.029	0.176
EDA	1	2	1	-.084	0.028	0.003	-0.139	-0.029
		3	1	-.078	0.029	0.008	-0.136	-0.020
		3	2	0.005	0.026	0.832	-0.045	0.056
	2	2	1	0.031	0.031	0.328	-0.031	0.092
		3	1	0.110	0.042	0.010	0.027	0.194
		3	2	0.080	0.028	0.005	0.025	0.135
EDANorm	1	2	1	-.256	0.101	0.013	-0.457	-0.055
		3	1	-.345	0.095	0.000	-0.534	-0.157
		3	2	-0.089	0.088	0.313	-0.264	0.085
	2	2	1	-0.039	0.045	0.389	-0.129	0.051
		3	1	0.037	0.056	0.512	-0.074	0.148
		3	2	0.076	0.042	0.071	-0.007	0.159

## Discussion

This study quantifies inconsistency in decision making for data empirically derived from participants in a controlled experiment. The focus on the influence of repeated trials on decision making inconsistency based on empirical data is a unique feature of this work because areas are poorly explored. The empiric origin of the data is rare and the general practice is to use randomly generated comparison matrices. A singular exception is the work presented in [30] which coincidentally also selects from a population of university students.

Our results reveal that if the decision problem is repeated, then the level of inconsistency depends on the problem size. In case of smaller problem sizes of up to five items, the inconsistency decreases as the decision task is repeated. Meanwhile, if the decision task involves comparing 6–10 items, then repetition of evaluation leads to an increase of the inconsistency level. It seems that for the larger matrices the inability to reduce the inconsistency was implied by the weaknesses of the applied 1-9 scale, which was not discriminative enough to allow for differentiating between so many criteria (in particular, that their subsets were already compared separately in different matrices). This weak point can represent one of future research directions as alternative scales and approaches have already been developed. For instance, the best–worst method (BWM) improves the AHP approach [40]. It changes the pairwise comparison from AHP into the comparison between the remaining criteria and the best–worst criteria [41]. Rezaei et al. [42] provide an example on a supplier selection. Comparison of application of AHP and BWM can shed light on the role of applied scale during inconsistency measurement.

Our findings expand the body of knowledge from the respective field of study because other authors investigate the influence of other factors on inconsistency in decision making. Importance of explanation of inconsistency in a set of propositions is revealed in [43]. The empirical findings show that once an explanation of inconsistency has been formulated by a participant, the participant is able to detect inconsistent assertions in a relatively low number of cases. When the decision making is performed in groups, shared information has an impact on the process, as shown by [44]. Preference-inconsistent shared information has a bigger impact on the decision when compared to the shared information, which is consistent with the preferences of the group.

The validity of the results of our work is circumscribed by the experiment settings. First, from the basic descriptive indicators perspective, such as gender or age, the analyzed sample of subjects is considered to be representative with respect to the defined population. However, the results associated with university students in a specific study field are difficult to generalize. Therefore, the experiment needs to be followed by further experiments with distinct target groups. Second, although a general decision-making domain was selected, the level of the participants’ expertise in the domain is unknown. Third, the results are only associated with one method of pairwise comparison. Therefore, the application of a wider range or higher granularity of available values may provide different results. The answers provided when filling smaller matrices affect the consistency of larger matrices in view of using a very specific 1-9 scale of AHP. With a smaller number of criteria, the user has a tendency of using a greater range of performances on a ratio scale than they would do for the same subset knowing there are more criteria, but the performance scale remains the same.

The experimental procedure was defined in a manner that allows its reproduction by other authors. Hence, further research can focus on other inconsistency indices applicable for inconsistency quantification, as mentioned e.g. by [23]. Furthermore, the effect of alternatives order preserving and not preserving experiment settings can be tested. Also, the order of alternatives can be set identically for all problem sizes. The order of presentation of problems of different sizes can be changed from gradually increasing to, for example, gradually decreasing, random, and partially ordered “big first, small last”. The design of the experiment strongly influenced the results. It is well known from the behavioural studies that there exists anchoring/adjustment effect [45], and hence the responses in the previous trials affect the responses in the following ones. Thus, if the subjects were not informed about the consistency rules of AHP before the first trial, the inconsistencies in their responses are propagated later on. The effect of fatigue and problem-solving environment presentation can also be examined. A specific decision-making domain or topic can also be defined. Although one can claim that each individual is an expert in a car selection problem, there is a quite low possibility of treating the involved students as real-world decision makers. The subjects did not have any chance of playing out their choices for real. Moreover, the experiment was based on the voluntariness of attendance. No incentives to students were provided. Some multi-criteria decision-making experimental studies like [46], which also involved students, adjusted both the problem and the incentives so that the students had a real interest in providing reliable answers. On the other hand, motivational theories from the field of psychology point out that any particular incentive can not guarantee honesty and endeavour of a group of people with non-zero level of heterogeneity.
